# Evaluating strategies to manage and endure challenging behaviors in mucopolysaccharidoses

**DOI:** 10.1186/s13023-021-01767-8

**Published:** 2021-04-08

**Authors:** Nathan Grant

**Affiliations:** Health, Medicine, and Society, University of Cambridge, Cambridge, UK

**Keywords:** Mucopolysaccharidoses, MPS, Challenging behavior, Aggression, Caregivers, Siblings, Coping, Support

## Abstract

The mucopolysaccharidoses (MPS) are a group of rare, genetic, lysosomal storage disorders characterized by progressive, multisystemic accumulation of glycosaminoglycans. Individuals with severe MPS often present with significant neurological involvement and may exhibit challenging behaviors, including hyperactivity, aggression, and sleep disturbance. These behaviors can cause adverse outcomes and necessitate the development of specific measures to support affected families. Through an analysis of the results reported by Hoffmann et al. in their recent study, this letter outlines important factors that must be considered when evaluating the impact of challenging behaviors associated with MPS, including treatment history, age, sibling and family relationships, the feasibility of daily caregiving, and caregiver burden. These recommendations can help guide future studies to identify the most effective coping strategies to support families of people with MPS who have challenging behaviors.

For many years, my family has struggled to provide care for my twin brother because of his challenging behaviors. My brother has the neuronopathic form of mucopolysaccharidosis (MPS) type II, also known as severe Hunter syndrome. Like many people with this condition, my brother is nonverbal, has a significant intellectual disability, and requires continual care and assistance from multiple caregivers [1, 2]. Although my parents and I assist my brother with daily living activities, including dressing, feeding, bathing, and toileting, it can be extremely difficult to provide care when my brother is agitated and aggressive. In the midst of changing my brother’s diaper or dressing him, we have to carefully watch my brother’s hands to ensure he does not punch us or pull our hair. Our attempts at self-defense are usually futile, however, leaving us with bruises running up our arms, scratches around our necks, torn clothes, and hair left scattered on the ground. Since my brother is nonverbal, it is difficult to ascertain the specific causes of his aggression and we have not found any strategies to effectively manage his challenging behaviors. My family lives in constant fear of my brother’s next aggressive outburst, uncertain whether he approaches us to hug us or to harm us. The recent study by Hoffmann et al. [3] provides important and timely insights on practical coping strategies to manage challenging behaviors in MPS. However, some additional questions and implications must be addressed in order to provide the most effective support for people with MPS and their caregivers.

Hoffmann et al. surveyed families of children diagnosed with MPS I, II, and III with serious neurological involvement. While the researchers collected some sociodemographic information about the children with MPS, such as their age and gender, they did not fully assess the children’s treatment history. Although Hoffmann et al. did report that one child had a history of MPS-specific therapy [3], it is unclear whether this therapy included enzyme replacement therapy (ERT), hematopoietic stem cell transplantation (HSCT), or another form of medical treatment. Furthermore, we are not provided information about the medical history of the other 33 children in the study. Medical treatment of any kind, including ERT, HSCT, anticonvulsants, pain management, treatment for spasticity, and surgeries, can significantly alter the behavioral manifestations of MPS [4]. For example, because many individuals with neuronopathic MPS have significant language impairment, they often exhibit challenging behaviors to communicate underlying medical issues, such as pain [4, 5]. Accordingly, an individual who has received more treatment for pain may exhibit fewer challenging behaviors than someone who has not been adequately treated for pain, which can significantly affect the frequency of behavioral symptoms as well as the efficacy of the coping strategies reported in this study. It would be truly devastating for a family to continually employ strategies to distract or calm an aggressive child when the behavioral antecedents are actually somatic in nature and could be effectively addressed with some form of medical intervention. Therefore, future studies must consider the medical history of people with MPS to thoroughly examine the cause of behavior problems and identify effective strategies for support.

While Hoffmann et al. provide important information about challenging behaviors in children under the age of 18, it is unfortunate that they did not include older participants in their study. Although the authors do not explicitly mention the reasons for this age limit, it is important to acknowledge that challenging behaviors still occur in people with MPS who are above the age of 18 [6]. The fact that two adults were excluded from the researchers’ analysis [3] indicates that there is both interest and a need to study challenging behaviors in older populations. In fact, it can be even more difficult to manage behavior problems in adults with MPS because of their physical size and strength [4, 7, 8]. Often multiple caregivers are needed, including parents, siblings, as well as home health aides and direct support professionals [2, 4]. However, the national shortage of caregivers and dismally low financial compensation for caregiving makes it difficult for families to provide care for adults with severe behavior problems [9]. With limited resources for support, many families must consider residential facilities when they are no longer able to provide care at home [4, 6]. Accordingly, future studies must investigate the experiences of families who provide care for adults with MPS in order to develop appropriate resources for support.

In addition to researching the experiences of adults with MPS, we must also pay attention to other members in the family. Hoffmann et al. report that MPS can have a slightly negative impact on relationships with healthy siblings [3]. However, of the 20 participants who responded to the question about sibling relationships, the majority of responses were from parents and only one questionnaire was actually completed by a sibling. Siblings may provide care for their brothers and sisters with MPS throughout life, and the role of primary caregiver often transitions to siblings as parents age [7]. Given the limited support available for siblings [7, 10], challenging behaviors may cause siblings to experience more emotional stress than parents and may necessitate unique coping strategies specifically for siblings. Accordingly, more research is needed to directly investigate the perceptions of siblings themselves. Researchers must work closely with patient and family organizations to invite siblings to participate in future studies.

Some of the coping strategies identified by Hoffmann et al. also raise concerns about practicality. Several measures reported against challenging behaviors require the direct involvement of caregivers, such as “calming child down,” “giving attention,” “rewarding/punishing/scolding child,” “1:1-supervision,” and “parent sleeping in child bed” [3]. While participants perceived many of these measures to be effective, these interventions all require time and physical presence. Yet Hoffmann et al. report that participants were better able to cope with their child’s disorder when they had time alone and outside the family [3]. These results seem conflicting: How do caregivers get time for themselves when they must constantly provide care and manage the behavior problems of MPS? Hoffmann et al. do not provide information about how participants feel actively implementing these coping strategies and their overall perceptions of caregiver burden. Therefore, qualitative studies are needed to examine whether these coping strategies are actually feasible in practice. Anthropological methods, such as participant observation, may be used to report how caregivers implement these strategies on a daily basis and identify whether these strategies should be recommended for all families.

Interestingly, Hoffmann et al. also report that antipsychotics were prescribed and rated highly effective against challenging behaviors [3]. However, previous studies have reported that antipsychotics should not be recommended for people with MPS because of their limited efficacy and potential for adverse side effects [4]. These conflicting results make it very difficult for clinicians and families to manage the behavior problems of MPS. Further qualitative research may help evaluate how antipsychotics affect the lived experiences of people with MPS, which may provide further insights on the efficacy of antipsychotics for this population.

While many factors can influence the impact of behavior problems, we must not forget that challenging behaviors can cause tremendous social isolation for families. My parents and I rarely see extended family, visit with friends, or go out in public because of fear that my brother will hurt someone. Moreover, support groups rarely discuss aggression and other behavior problems, making it seem like we are the only family with these difficult experiences. The research conducted by Hoffmann et al. is extremely important because it helps families like mine recognize that we are not alone and there are strategies to help cope with challenging behaviors. However, the recommendations outlined in this article can help studies dig even deeper to understand the broader impact of challenging behaviors and identify the most effective interventions for support.

## Data Availability

Not applicable.

## Abbreviations

MPS: Mucopolysaccharidosis
ERT: Enzyme replacement therapy
HSCT: Hematopoietic stem cell transplantation
