# Supplementary material for: Evaluating strategies to manage and endure challenging behaviors in mucopolysaccharidoses
Source: Orphanet J Rare Dis. 2021 Apr 8;16:165. doi: 10.1186/s13023-021-01767-8 (PMC8033673; doi:10.1186/s13023-021-01767-8)
Supplement: Related texts

# Reply: Response to: Evaluating strategies to manage and endure challenging behaviors in mucopolysaccharidoses

**Authors:** Frederik Hoffmann, Markus Ries

**Affiliations:** 2 grid.492024.9 0000 0004 0558 7111 Center for Pediatric and Adolescent Medicine, Klinikum Fürth, Jakob-Henle-Straße l, 90766 Fürth, Germany; 3 grid.5253.1 0000 0001 0328 4908 Pediatric Neurology and Metabolic Medicine, Center for Rare Diseases, Center for Pediatric and Adolescent Medicine, Heidelberg University Hospital, Im Neuenheimer Feld 430, 69120 Heidelberg, Germany

Dear Professor Palau,

Thank you for letting us have Grant’s response to our research. We have studied Grant’s letter with great interest and certainly appreciate his important comments. We feel honoured by such scholarly feedback and are happy to comment on it.

Grant strongly underlines the urgent need for a better understanding of the behavioral problems associated with neuronopathic Mucopolysaccharidoses (MPS) and their deep-reaching effects on affected families, as well as the problems in surveying them. He concretely illustrates how aggressive behavior further impedes the intense and stressful demands on the people caring for persons with neuronopathic MPS. His experiences conform with the findings of our study, which pinpoint out aggressive behavior as one of the most wearing and least manageable problems associated with this disorder. Furthermore, the social isolation that results from the challenging behavior is a core problem with MPS, as it reduces external support resources. Accordingly, parents have reported an open exposure to the disorder and its difficulties as advice to ‘recently diagnosed’ families [3]. We agree with Grant, that the awareness of affected families’ situation and the removal of taboos seems a cutting-edge element in the commerce with the disease.

Against the background of patients being intellectually disabled and nonverbal, as emphasized in our publication, behavior such as aggression must be considered as communication of indisposition [3, 11]. We have mentioned that it is important to address potential physical factors first, in order to allow for behavioral strategies to work out [3, 4]. In this context, availability by a practitioner, who is familiar with the child and its condition, seems crucial. Parents did not report the use of pain killers in our study [3]. However, in our clinical experience we have seen the positive effects of antispastic, hence analgetic treatment via baclofen pump on sleep difficulties.

We conform with Grant that the individual medical history and medical treatment is fundamental for understanding child behavior and possible solutions. Yet, a wide collection of data will decrease study participants’ response rates (a factor that must not be underestimated in research intense conditions like MPS). Therefore, the study design needed to balance these factors in order to answer our research questions. Our primary interest focused on the behavioral phenotype and the collection of parental experiences. With this aim in mind, all medication prompted by parents was presented in the publication, each with its estimated effectiveness on difficult behavior. The high reporting rate of antipsychotic medication shows, that its prescription is common in the treatment of sleep disturbance and hyperactivity in MPS [3]. We have underlined that antipsychotic medication may have strong adverse effects, especially in children with MPS, and must therefore be prescribed with particular precaution [3, 4, 12–14]. However, given its high estimated effectiveness on challenging behavior, the use of antipsychotic medication should not be dismissed entirely without careful specialist evaluation. An individual (re-)consideration of risks and benefit before and after the beginning of such treatment is considered inevitable and severe adverse effects may call for a discontinuation of treatment. Medication should be evaluated in collaboration with a pediatric psychiatrist. However, regional availability of such medical support is often limited and represents a resource constraint.

Although Enzyme Replacement Therapy (ERT) or Hematopoietic Stem Cell Transplantation (HSCT) can have an influence on the disease trajectory, its use is limited to a minority of affected children [15–18]. In our study’s population only one child had received such specific therapy [3]. Due to the rarity of the disorder, the child’s mother feared to endanger her anonymity. We respected her wish for confidentiality and did not state the specific therapies her child had received prior to the study. Nevertheless the individual treatment history is essential for the understanding of child behavior in MPS.

Symptomatic behavior in MPS shows distinct changes over time. Hyperactivity, as one of the core symptoms in children, would diminish in the process of ongoing neuronal impairment and ultimately lead to a vegetative state in most patients [19–21]. Our study focused on the pediatric behavioral phenotype and referring challenges, which lead to the exclusion of adult individuals in our study protocol. We are thankful for Grants accentuation, that care demands do not end with the age of 18 years but shift towards other challenges. This being so, caregiving will tend to be hindered by adult patients’ physical strength, whilst overall patient activity declines. Again, siblings may be called on to take responsibility. This underlines the importance of external support for affected families in a post-pediatric population. We agree with Grant, that studies investigating challenges of care demands in adult patients with MPS are urgently needed.

Our questionnaire addressed caregivers of children with MPS. The fact that it was answered mostly by mothers may be representative for mothers being primary caregivers in MPS [8]. Grant noted that sibling stress can’t be directly measured in our study. In fact, in only one of 34 cases, a sibling answered our questionnaire herself. As illustrated in previous studies, siblings of children with chronic conditions rather internalize their stress, trying not to further burden their families with their own problems [11, 22, 23]. Therefore studies directly addressing siblings are needed. Furthermore programs to support, connect and empower siblings of chronically ill children are highly important [24].

Patient care demands high mental and physical efforts [8, 11, 25]. Nevertheless, affected families must not neglect their own wellbeing and recovery, in order to prevent burnout and master ongoing care demands. Grant highlights this dilemma by raising concerns about the practicability of time-consuming measures. The timely and psychological efforts accompanying any coping measure is highly individual and depends on multiple factors of the patient (degree of disability, extent of challenging behavior such as aggression) and caregiver (physical and mental robustness, resources) [11]. In our study, we have tried to gather parental experiences and state possible toeholds in the contact with affected children. The evaluation of cost and benefit of specific measures must be individual and curtailed to distinct needs and resources in order to find a balance that allows for long-standing care. The conflict of care demands and recovery underlines the importance of external support for affected families.

The findings of our study may serve as a first step towards using parental experience with problems of poor manageability. Future studies, ideally in a controlled study design, are urgently needed to monitor efficacy, safety, practicability and use of specific measures.
